# Efficacy-Based Perspective to Overcome Reduced Opioid Analgesia of Advanced Painful Diabetic Neuropathy in Rats

**DOI:** 10.3389/fphar.2019.00347

**Published:** 2019-04-09

**Authors:** Mihály Balogh, Ferenc Zádor, Zoltán S. Zádori, Mohammed Shaqura, Kornél Király, Amir Mohammadzadeh, Bence Varga, Bernadette Lázár, Shaaban A. Mousa, Sándor Hosztafi, Pál Riba, Sándor Benyhe, Klára Gyires, Michael Schäfer, Susanna Fürst, Mahmoud Al-Khrasani

**Affiliations:** ^1^Department of Pharmacology and Pharmacotherapy, Faculty of Medicine, Semmelweis University, Budapest, Hungary; ^2^Institute of Biochemistry, Biological Research Centre of the Hungarian Academy of Sciences, Szeged, Hungary; ^3^Department of Anaesthesiology and Intensive Care Medicine, Charité University Berlin, Berlin, Germany; ^4^Department of Pharmaceutical Chemistry, Semmelweis University, Budapest, Hungary

**Keywords:** diabetes, neuropathic pain, opioid efficacy, 14-*O*-methylmorphine-6-*O*-sulfate, morphine, fentanyl

## Abstract

Reduction of the opioid analgesia in diabetic neuropathic pain (DNP) results from μ-opioid receptor (MOR) reserve reduction. Herein, we examined the antinociceptive and antiallodynic actions of a novel opioid agonist 14-*O*-methymorphine-6-*O*-sulfate (14-*O*-MeM6SU), fentanyl and morphine in rats with streptozocin-evoked DNP of 9–12 weeks following their systemic administration. The antinociceptive dose-response curve of morphine but not of 14-*O*-MeM6SU or fentanyl showed a significant right-shift in diabetic compared to non-diabetic rats. Only 14-*O*-MeM6SU produced antiallodynic effects in doses matching antinociceptive doses obtained in non-diabetic rats. Co-administered naloxone methiodide (NAL-M), a peripherally acting opioid receptor antagonist failed to alter the antiallodynic effect of test compounds, indicating the contribution of central opioid receptors. Reduction in spinal MOR binding sites and loss in MOR immunoreactivity of nerve terminals in the spinal cord and dorsal root ganglia in diabetic rats were observed. G-protein coupling assay revealed low efficacy character for morphine and high efficacy character for 14-*O*-MeM6SU or fentanyl at spinal or supraspinal levels (*E*_max_ values). Furthermore, at the spinal level only 14-*O*-MeM6SU showed equal efficacy in G-protein activation in tissues of diabetic- and non-diabetic animals. Altogether, the reduction of spinal opioid receptors concomitant with reduced analgesic effect of morphine may be circumvented by using high efficacy opioids, which provide superior analgesia over morphine. In conclusion, the reduction in the analgesic action of opioids in DNP might be a consequence of MOR reduction, particularly in the spinal cord. Therefore, developing opioids of high efficacy might provide analgesia exceeding that of currently available opioids.

## Introduction

Neuropathic pain (NP) is a chronic pain condition that limits patients to fully achieve their daily tasks. Consequently, NP has significant impact on the economic welfare of the society (Bouhassira et al., 2008; Gaskin and Richard, 2012). Therefore, to find drugs satisfactorily treating NP is a major clinical goal. The management of severe acute to moderate pain including cancer pain can be achieved by opioids, however, opioid effectiveness in the treatment of chronic NP is controversial (Wiffen et al., 1996; Eriksen et al., 2006; Furlan et al., 2006; Derry et al., 2016; Hoffman et al., 2017). At present, opioids are considered as a second-to-third-line medication for NP. However, they can be considered as first line drugs, when they may offer advantages over the general first line drugs for instance in case of cancer NP, an acute pain attack or during first line drug titration. Data from *in vivo* experimental studies have reported on a significant reduction in opioid antinociceptive efficacy in neuropathic animals following systemic administration (Courteix et al., 1998; Hama and Sagen, 2007). The loss in opioid antinociceptive efficacy has also been reported following central (spinal or supraspinal) administration in NP animals (Ohsawa and Kamei, 1997; Zurek et al., 2001). It was proposed that the impaired opioid antinociception occurred as a consequence of the decrease in opioid receptor reserve (Zhang et al., 1998). In addition, the reduction in opioid receptor density in spinal tissues was also demonstrated in diabetic animals (Courteix et al., 1998; Shaqura et al., 2013).

Based on these observations, we hypothesize that the reduction in MOR number in diabetic neuropathic pain (DNP) is a crucial factor in the loss of analgesic effect of opioids. Recently, we have demonstrated that opioids of high efficacy in contrast to morphine could produce significant maximal effects (efficacy) in isolated organs hosting low opioid receptor reserve (Al-Khrasani et al., 2007; Riba et al., 2010; Lacko et al., 2012; Khalefa et al., 2013; Zádor et al., 2017).

Thus, opioids of high intrinsic efficacy displaying spare receptors might produce analgesic effects even though the MOR reserve is low. In the past two decades our group has paid attention to the efficacy of opioids, particularly to MOR agonists (Al-Khrasani et al., 2007, 2012; Lacko et al., 2012; Kiraly et al., 2015; Lackó et al., 2016; Zádor et al., 2017; Balogh et al., 2018). These previous studies have proved the high efficacy character for 14-*O*-methylmorphine-6-*O*-sulfate (14-*O*-MeM6SU).

Therefore, the present study was aimed to examine the antinociceptive and antiallodynic effects of the recently synthetized 14-*O*-MeM6SU compared to the high efficacy fentanyl and low efficacy morphine in a rat model of DNP. To this extent, the experiments were carried out 9 and 12 weeks following intraperitoneal (i.p.) streptozocin (STZ) treatment. The number of MOR was also evaluated in dorsal root ganglia (DRG) and dorsal horn tissues of diabetic and non-diabetic rats. Finally, we measured G-protein coupled receptor activity to determine the efficacy of test compounds in diabetic rat spinal cord and brain homogenates. Prior to *in vivo* and *in vitro* experiments, the change in the paw threshold, blood glucose level, weight, as well as water- and food consumption of diabetic and control rats was followed.

## Materials and Methods

### Animals

Male Wistar rats of 200–300 g were used for STZ-induced diabetes model. The animals were obtained from the local Animal House (Semmelweis University, Budapest, Hungary). The animals were kept in mesh bottomed cage (4, 5, or 6 animals/cage depending on the weight of animals) in a room of 20 ± 2°C temperature, 12-h/12-h light/dark cycle, in the local animal house of the Semmelweis University, Department of Pharmacology and Pharmacotherapy (Budapest, Hungary). The type of the cage was eurostandard type IV. The floor area is 1820 cm^2^. This type allows to keep 7 rats of 300 g according to EU recommendation. Water and standard food were available *ad libitum*. In the first series of experiments control (vehicle treated) and diabetic (STZ treated) animals were kept individually in mash bottomed cages making the measurement of individual parameters possible.

All housing and experiments were performed in accordance with the European Communities Council Directives (2010/63/EU), the Hungarian Act for the Protection of Animals in Research (XXVIII.tv. 32.§) and local animal care committee (PEI/001/276-4/2013). All the researchers did the best effort to minimize the number of animals and their suffering.

### Chemicals

The morphine analog 14-*O*-methylmorphine-6-*O*-sulfate (14-*O*-MeM6SU) was provided by the Department of Pharmaceutical Chemistry, Semmelweis University (Budapest, Hungary) and was synthesized and characterized as previously described (Lacko et al., 2012). Naloxone methiodide (NAL-M), STZ and clonidine-hydrochloride were obtained from Sigma-Aldrich Ltd. (Budapest, Hungary) and morphine hydrochloride from Alkaloida-ICN (Tiszavasvári, Hungary). Fentanyl was purchased from Toronto Research Chemicals (North York, ON, Canada), Tris-HCl, EGTA, NaCl, MgCl_2_ x 6H_2_O, GDP, the GTP analog GTPγS, were purchased from Sigma-Aldrich Ltd. (Budapest, Hungary). The MOR agonist enkephalin analog Tyr-D-Ala-Gly-(NMe)Phe-Gly-ol (DAMGO) was obtained from Bachem Holding AG (Bubendorf, Switzerland). Ligands were dissolved in water and were stored in 1 mM stock solution at -20°C for *in vitro* tests. Ligands used for *in vivo* assays were dissolved in saline or in the case of STZ ice cold distilled water prior to the experiments.

The radiolabeled GTP analog, [^35^S]GTPγS (specific activity: 1000 Ci/mmol) was purchased from Hartmann Analytic (through Izotóp Intézet Kft., Budapest, Hungary). The UltimaGold^TM^ MV aqueous scintillation cocktail was purchased from PerkinElmer (through Per-Form Hungária Kft., Budapest, Hungary). All compounds were stored and handled as described in the product information sheet.

### Streptozocin (STZ)-Induced Diabetic Neuropathic Pain Model (Model of Polyneuropathy)

#### Experimental Design

**FIGURE 1:**
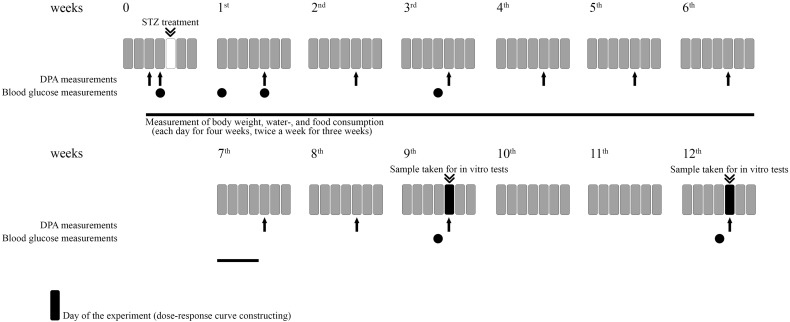
Representative figure illustrating the experimental protocol. For each experiment different animals were used. For *in vitro* studies different groups were used.

The experimental procedures for experiments designed to assess the induction of hyperglycemia, allodynia, polydipsia, polyphagia, weight change as well as the assessment of drug antinociception are included in Figure 1.

#### Induction of Diabetes

We used STZ to induce diabetes. The animals were treated i.p. with 60 mg/kg streptozocin in a 2.5 ml/kg volume. The STZ was diluted in cold distilled water as described previously and the solution was made less than 10 min before the injection to avoid any degradation (Courteix et al., 1994; Rajaei et al., 2013). Vehicle treated group was used as absolute control.

#### Measuring the Blood Glucose Levels of Diabetic Animals

Blood glucose level was determined prior to and 72 h, 1, 3, 9, and 12 weeks after STZ or vehicle treatments. Animals were slightly anesthetized with 3% isoflurane in oxygen via nose cone as described previously (Balogh et al., 2018). Then one drop of blood was taken from the tail veins. Accu-Chek Active blood glucose meter (Roche Diagnostics GmbH, Germany) was used to measure blood glucose levels. The maximal measurable blood glucose level value by blood glucose test is 33.3 mmol/l. Animals with blood glucose level of 14 mmol/l or higher were considered diabetic as described previously (Courteix et al., 1993).

#### Measuring of Animal Weight and the Consumption of Food and Water of Diabetic Animals

In the first series of experiments control (vehicle treated) and diabetic (STZ treated) animals were kept individually. Then, the water and food consumption were measured separately for each animal before and after the STZ treatment during 7 weeks (Figure 1). In this period, the weight of the food and water was measured each day for 4 weeks and two times a week for 3 weeks. The consumed amount was calculated. The weight of the animals were also measured during the 7 weeks period in the same way and on the day of experiments. Thereafter, animals were kept in groups until the day of experimentation.

#### Determination of Gastric Emptying in Diabetic and Non-diabetic Rats

Gastric emptying was assessed by the phenol red method as described earlier (Fülöp et al., 2005), with some minor modifications. Briefly, after 24 h of fasting, diabetic and non-diabetic rats received 1.5 ml of 1.5% methylcellulose solution containing 0.5 mg/ml phenol red (a non-absorbable marker compound) by intragastric gavage. After 20 min the rats were sacrificed and the stomachs were removed after clamping the pylorus and cardia. The content of stomach was mixed with 40 ml of 0.1 N NaOH, then 0.6 ml mixture was added to 1.2 ml of 7.4% trichloroacetic acid solution to precipitate proteins. After centrifugation (15 min, 3000 g) 1.2 ml of the supernatant was added to 0.6 ml of 1 N NaOH, and the absorbance was read spectrophotometrically in triplicates at 560 nm. Gastric emptying (%) was calculated as follows: [1 – (absorbance of sample / maximal absorbance)] × 100. Maximal absorbance was measured by processing the test meal alone, as described above.

In a separate experiment, weight match animals were treated either with saline, or with clonidine (0.1 mg/kg), an alpha_2_ adrenoceptor agonist and well-known inhibitor of gastric emptying, subcutaneously 30 min before the methylcellulose administration.

#### Assessment of Neuropathic Pain by Dynamic Plantar Aesthesiometer (DPA)

In order to determine the allodynia caused by advanced diabetes we used the DPA (Ugo Basile, Italy) as described previously (de Novellis et al., 2011) with slight modifications based on our the pilot experiments. The animals were placed in the plastic cages of the DPA once daily for 3 subsequent days (“handling”). Before each experiment, animals were kept in these cages for at least 5 min before starting the measurement in order to habituate them. The equipment raises a straight metal filament of 0.5 mm diameter until it touches the hind paws. Then it puts pressure on the paw with an increasing force from 1 to 50 g (cut off). The maximal force applied by DPA is 50 g (as prescribed by manufacturer instrument guide).

The paw pressure withdrawal thresholds (PPTs) were measured and expressed in g, before and after the STZ treatment on every 3rd week. In the first series of experiments, PPTs were measured weekly after STZ treatment in order to detect touch allodynia. PPTs of each hind paw were measured 3 times alternately. Then, the average of PPT values of the two paws were calculated for each animal. Vehicle treated and weight match (i.e., animals with weights matching the diabetic ones) groups were used as control. After determination of the time of peak effect further analysis was made at that time point. An animal was considered neuropathic, when the PPT value was at least decreased by 20% compared to weight match animals as described previously (Király et al., 2018).

It is important to note that significant change in the weight of animals result in significant alteration in the PPTs (i.e., higher weight elevates the threshold values, data not shown). This weight change may contribute to the potential differences observed between control and diabetic animals in PPTs. Therefore, to justify this alteration, the use of weight match control animals was necessary, to have a more exact analysis as also described previously (Végh et al., 2017). Weight match animals were handled and kept under the same conditions described for the diabetic (STZ treated) and non-diabetic control (vehicle treated) animals. The exception is that weight match animals were kept only for at least 1 week prior to experiments. In addition, the weight matched animals were also used to avoid lack of blindness during the experiments, though the symptoms of diabetes can not be fully masked.

#### Treatment of Neuropathic Animals

The test compounds were dissolved in saline in a volume of 2.5 ml/kg body weight. Baselines of PPTs were measured before subcutaneous (s.c.) agonist treatment. The opioid antagonist, NAL-M was co-administered with morphine and 14-*O*-MeM6SU. Solutions were prepared right before the experiment (in less than 10 min). The experiments were randomized and the experimenter was blind to the treatments.

### Radio-Ligand Binding Assays

#### Membrane Preparations

Membranes were obtained from lumbar dorsal root ganglions (DRGs) and spinal cord (L3-5), quickly frozen, and dissected into the ventral and dorsal half, as described previously (Shaqura et al., 2016; Mousa et al., 2017). DRGs and only the dorsal part of the spinal cord were further processed for radioligand binding. The tissues were placed immediately in ice cold assay buffer (50 mM Tris-HCl, 1 mM EGTA, 5 mM MgCl_2_, pH 7.4), homogenized with a Polytron homogenizer (Kinematica, Littau, Switzerland), and centrifuged at 48,000 *g* at 4°C for 20 min. The pellets were resuspended in assay buffer followed by 10 min incubation at 37°C to remove endogenous ligands. The homogenates were centrifuged again at 48,000 *g* and resuspended in assay buffer. Membranes were aliquoted and stored at -80°C.

#### Opioid Receptor Binding

Specific binding of [^3^H]DAMGO was performed by incubating 50–100 μg of membrane protein of lumbar dorsal horn with 0.1- 4 nM [^3^H]DAMGO in the presence or absence of 10 μM unlabelled naloxone to determine non-specific binding. Membranes were incubated for 1 h at 22°C in assay buffer. The reactions were terminated by rapid filtration under vacuum through Whatman GF/B glass fiber filters, followed by four washes with cold buffer (50 mM Tris–HCl, pH 7.4). Bound radioactivity was determined by liquid scintillation spectrophotometry (Perkin Elmer, Rodgau, Germany) at 60% counter efficiency after overnight extraction of the filters in 3 ml of scintillation fluid. All experiments were performed in duplicate and carried out at least five times. *B*_max_ and *K*_d_ values in saturation binding assays were determined by nonlinear regression analysis of concentration-effect curves using GraphPad Prism (GraphPad Software Inc., San Diego, CA, United States).

### Immunohistochemistry

Rats were deeply anesthetized with isoflurane and transcardially perfused with 100 ml of phosphate buffered saline (PBS) pH 7.4, then followed by 500 ml of 4% (w/v) paraformaldehyde in phosphate buffer pH 7.4. After perfusion, DRG and spinal cord were removed from treated and control animals, postfixed in the same fixatives for 90 min, and then cryo-protected overnight at 4°C in PBS containing 10% sucrose. DRGs (10 μm thick) were mounted onto gelatin coated slides. DRG mounted or spinal cord floating tissue sections were incubated with the following primary antibody rabbit polyclonal MOR antibody (1:1,000, Gramsch Laboratories, Schwabhausen, Germany). The tissue sections were washed with PBS prior to incubation with Alexa Fluor 594 donkey anti-rabbit secondary antibody (Invitrogen, Germany). Finally, the tissues were washed in PBS, mounted on vectashield (Vector Laboratories, Burlingame, CA, United States) and viewed under Zeiss LSM 510 laser scanning microscope (Carl Zeiss, Göttingen, Germany). To demonstrate specificity of staining, the following controls were included as mentioned in detail elsewhere (Endres-Becker et al., 2007; Mousa et al., 2013): (1) Preabsorption of the primary antibody against MOR was verified by preabsorption with 5 μg/ml of synthetic peptide antigen for MOR (Gramsch Laboratories, Germany), for 24 h at 4°C; (2) Omission of either the primary antisera or the secondary antibodies.

#### Quantification of Immunostaining

The method of quantification of DRG and spinal cord dorsal horn immunostaining has been described in detail elsewhere (Shaqura et al., 2013). Briefly, the total number of MOR-IR neurons was counted in each area (320 μm^2^) and this number was divided by the total number of neurons in each DRG within the same area and represented as percentages. For quantification of MOR immunoreactivity in the dorsal horn of spinal cord images of red immunofluorescence were obtained using a Zeiss LSM 510 laser scanning microscope and the image-analysis software package 2.5 SP2 from Zeiss was applied to quantify changes in immunodensities as described in detail elsewhere (Shaqura et al., 2013). The settings of the confocal microscope were established using a control section and kept unchanged for all subsequent acquisitions. Six to eight images were sampled per animal. Images were thresholded to exclude background fluorescence and gated to include intensity measurements only from positively stained nerve fibers. For image analysis, a standardized area was positioned over the Rexed laminae 1-5 of all groups to determine the mean product of the area (100 μm^2^) and mean intensity of pixels within the threshold value and to calculate the integrated optical intensity (product of area and mean intensity). The mean value of control was considered as 100%. Five rats per group were used for analysis. Data were expressed as means ± SEM. Scale bar = 20 μm for DRG and 40 μm for spinal cord section.

### G-Protein Activity Assays

#### Membrane Preparations

Rats were decapitated and their brains and whole spinal cords were quickly removed and were prepared for receptor binding assays as previously reported (Benyhe et al., 1997; Zádor et al., 2014). In brief, first the brain and spinal cord were homogenized, centrifuged in ice-cold 50 mM Tris-HCl (pH 7.4) buffer and incubated at 37°C for 30 min in a shaking water-bath (for details see Benyhe et al., 1997). After incubation the centrifugation was repeated as described before and the final pellet was suspended in ice-cold TEM (Tris-HCl, EGTA, MgCl_2_) buffer and stored at -80^°^C for further use.

#### Functional [^35^S]GTPγS Binding Assays

In [^35^S]GTPγS binding experiments the GDP→GTP exchange of the G_αi/o_ protein by a radioactive, non-hydrolysable GTP analog, [^35^S]GTPγS is measured. The nucleotide exchange is measured in the presence of a given ligand in increasing concentrations to measure ligand potency and the maximal efficacy (Strange, 2010).

The functional [^35^S]GTPγS binding experiments were performed as previously described (Sim et al., 1995; Traynor and Nahorski, 1995), with some modifications. Briefly, the rat brain and spinal cord membrane homogenates containing ∼10 μg/ml protein were incubated at 30°C for 60 min in Tris-EGTA buffer (pH 7.4) composed of 50 mM Tris-HCl, 1 mM EGTA, 3 mM MgCl_2_, 100 mM NaCl. The incubation mixture contained 0.05 nM [^35^S]GTPγS, increasing concentrations (0.1 nM–10 μM) of 14-*O*-MeM6SU, fentanyl or morphine and excess GDP (30 μM). The final volume of the incubation mixture was 1 ml.

Total binding was measured in the absence of the test compounds, while non-specific binding was determined in the presence of 10 μM unlabeled GTPγS. The bound and unbound [^35^S]GTPγS were separated by rapid filtration under vacuum (Brandel M24R Cell Harvester), and washed three times with 5 ml ice-cold 50 mM Tris-HCl through Whatmann GF/B glass fibers (GE Healthcare Life Sciences through Izinta Kft., Budapest, Hungary). The radioactivity of the filters was detected in UltimaGold^TM^ MV aqueous scintillation cocktail with Packard Tricarb 2300TR liquid scintillation counter. [^35^S]GTPγS binding experiments were performed in triplicates and repeated at least three times.

### Statistical Analysis

#### *In vivo* Assays

For the analysis of mechanical pain thresholds of diabetic animals one-way or two-way ANOVA followed by Newman–Keuls *post hoc* test was applied. Likewise, in the case of weight change, water- and food consumption.

Dose response curves: dose response curves of percent inhibition of nociceptive response were constructed for each compound in rats 9 weeks after STZ treatment. Also dose response curves of percent inhibition of nociceptive response were calculated in weight matched non-diabetic rats for each tested dose of test compounds. The percentage change of PPT was determined in diabetic and weight matched non-diabetic rats as follows: effect (%) = (PPT_(*after treatment*)_ - PPT_(*before treatment*)_)/PPT_(*before treatment*)_.

Then, the effective dose producing 30% effect (ED30) was calculated from log-linear regression for each test compound in diabetic and weight matched non-diabetic rats. To analyze the changes in antinociceptive potency of test compounds, the calculated ED30 values were compared (ED30_diabetic_/ED30_non-diabetic_).

The antiallodynic effects of test compounds were analyzed by one-way ANOVA followed by Newman–Keuls *post hoc* test at the 9th and 12th week. Weight match animals were used as absolute control in DPA measurement. Vehicle treated group was used as control in order to decide if the applied treatment significantly influenced the parameters.

Results were considered statistically significant when *P* < 0.05. All the analyses were performed with a professional statistical software: GraphPad Prism 6.0 (GraphPad Software Inc., San Diego, CA, United States).

#### G-Protein Activity and Radioligand Binding Assays

The specific binding of [^35^S]GTPγS was calculated by the subtraction of non-specific binding from total binding and was given in percentage. Data was normalized to total specific binding, which was settled 100% and also represents the level of basal activity of the G-protein. Experimental data were presented as means ± SEM in the function of the applied ligand concentration range in logarithm form. Points were fitted with the professional curve fitting program, GraphPad Prism 5.0 (GraphPad Prism Software Inc., San Diego, CA, United States), using non-linear regression, applying the ‘Sigmoid dose-response’ equation to determine the maximum G-protein efficacy (*E*_max_) and ligand potency (EC_50_).

For the analysis of *E*_max_ values and the comparison of individual concentration points within the concentration curves two-way ANOVA with uncorrected Fisher’s LSD *post hoc* test was applied. Statistical analysis was performed with GraphPad Prism 6.0 program; significance was accepted at *P* < 0.05 level.

## Results

### The Development of Diabetic Symptoms and Neuropathic Pain (Allodynia) in STZ Treated Rats

**FIGURE 2:**
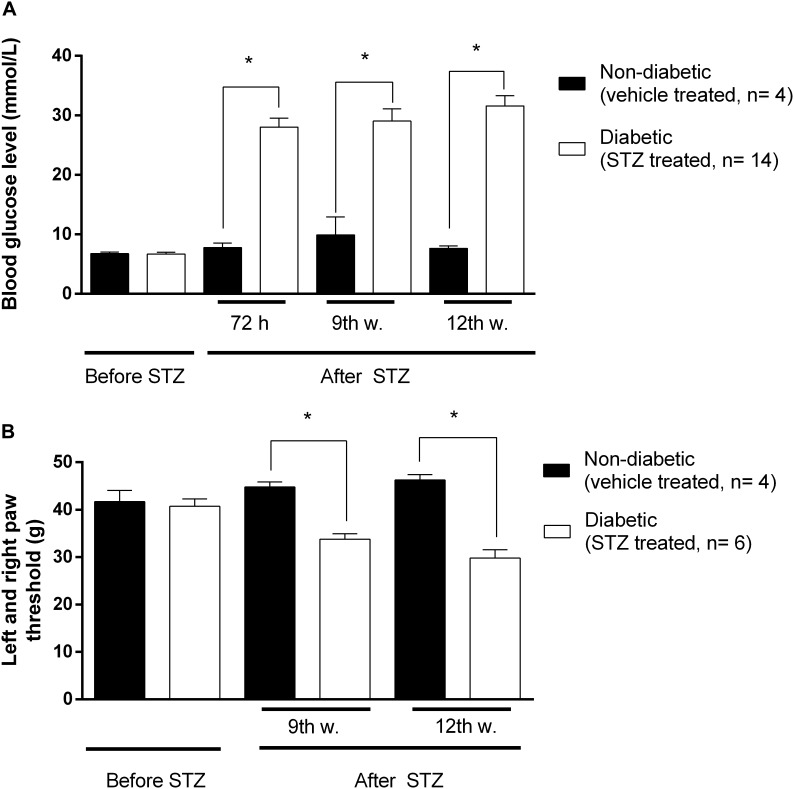
The changes in blood glucose levels in mmol/ml **(A)**, and hind paw withdrawal thresholds **(B)** in grams prior to and after STZ- or vehicle treatments. Animals not matching predefined criteria for diabetic neuropathy were excluded from experiments (at least 14 mmol/ml blood glucose level and at least 20% decrease in PPT compared to weight matched animals). Figure represents data from one series of experiments. Each value represents the mean ± SEM. ^∗^*p* < 0.05 vs. the signed groups (one way ANOVA followed by Newman–Keuls *post hoc* test).

Significant increase in blood glucose concentration of STZ-treated rats compared to vehicle treated animals was achieved 72 h following intraperitoneal STZ injections. This hyperglycaemia was maintained during the entire experimental period (9-12 weeks) indicating the development of diabetes (Figure 2A).

Also significant decrease in PPTs was achieved at the 3rd week following STZ injection indicating the development of mechanical allodynia (Figure 2B). This symptom is a key feature in the diagnosis of neuropathic pain. Figure 2 depicts that at the 9th and 12th week, diabetic animals developed the lowest nociceptive thresholds that were significantly lower compared to the baseline measured prior to STZ-treatment, indicating the peak of allodynia. No significant difference in developed allodynia was observed between the 9th and 12th week following STZ-treatment. Therefore, in our subsequent studies the antinociceptive action of test compounds, as well as MOR functioning, were analyzed 9 and 12 weeks after induction of diabetes.

Water intake of STZ treated rats was significantly increased in comparison with the vehicle treated group 48 h following treatment. The food consumption of rats with hyperglycaemia reached a significant increase 5 days after treatment (Supplementary Figure 1). Diabetic rats gained significantly less body weight compared to age matched animals. Therefore, weight matched non-diabetic rats were used for a comparison in nociceptive thresholds. In addition, we found no differences between the rates of gastric emptying in 12-weeks diabetic (80 ± 2%, *n* = 23) and non-diabetic rats (82 ± 2%, *n* = 20), whereas 0.1 mg/kg clonidine, an alpha_2_-adrenoceptor agonist used as a positive control, markedly delayed the emptying in weight-matched control animals (55 ± 2%, *n* = 7, *p* < 0.01 vs. saline-treated rats).

### Advanced Diabetes Impairs the Antinociceptive Effect of Systemic Morphine but Not 14-*O*-MeM6SU or Fentanyl in Rats

Prior to evaluation of the antinociceptive and anti-allodynic effects, the peak antinociceptive effects of 14-*O*-MeM6SU and morphine were established (60 min for 14-*O*-MeM6SU, 10 min for fentanyl and 30 min for morphine; Supplementary Figure 2). Thus, these times of peak effects were chosen for further analysis in the entire pain study by DPA.

**FIGURE 3:**
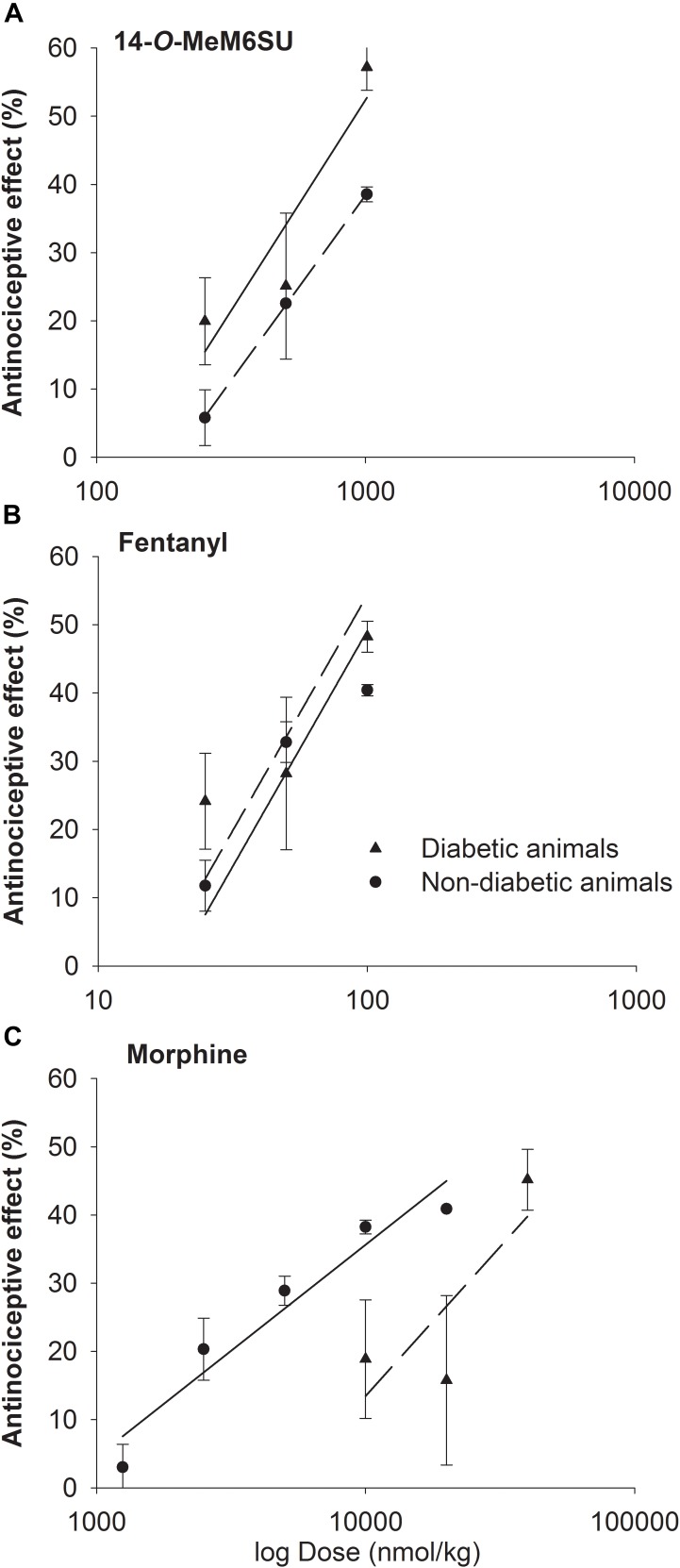
Dose-response curves of 14-*O*-MeM6SU **(A)**, fentanyl **(B)** and morphine **(C)** in diabetic and non-diabetic animals obtained with DPA. Data are represented as mean ± SEM (*n* = 5–10).

The calculated ED30 values of 14-*O*-MeM6SU were 434 and 335 nmol/kg for diabetic and non-diabetic animals, respectively. The ED30 values of fentanyl were 41 and 54 nmol/kg in the same order. In the case of morphine the ED30 values were 20692 and 6589 nmol/kg for diabetic and non-diabetic animals, respectively. Based on the calculated ED30 values there was no significant change in the antinociceptive effect of 14-*O*-MeM6SU or fentanyl (the value of ED30_diabetic_/ED30_non-diabetic_ was 1.3 for both compounds). On the other hand, morphine was 7 times less effective in diabetic, than non-diabetic animals (ED30_diabetic_/ED30_non-diabetic_) (Figure 3). These data also indicate that 14-*O*-MeM6SU displayed a 48 times and fentanyl 505 times higher potency than morphine in diabetic conditions based on the compared ED30 values.

### The Antiallodynic Effects of Systemic 14-*O*-MeM6SU, Fentanyl and Morphine in Diabetic Rats

The present data were obtained 9 and 12 weeks following STZ treatment that is 6 and 9 weeks after the significant appearance of allodynia, a major sign of painful diabetic neuropathy.

**FIGURE 4:**
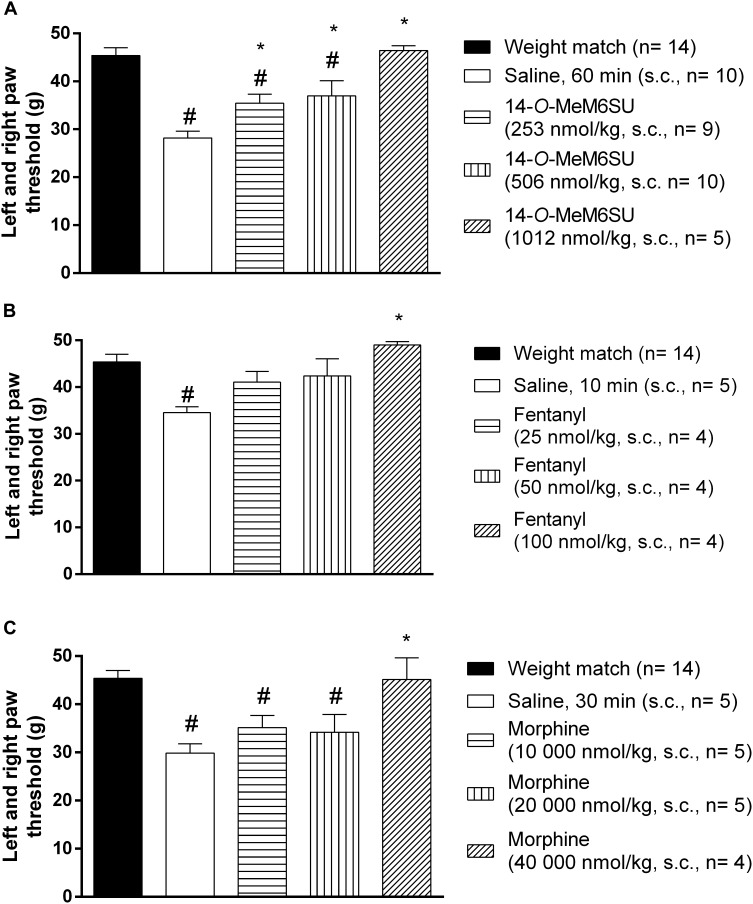
The systemic antinociceptive effect of 14-*O*-MeM6SU **(A)**, fentanyl **(B)** and morphine **(C)** in STZ treated diabetic rats with neuropathy on DPA test following systemic (s.c.) administration at 9th week after STZ injection. Data were obtained 60 min after the injection of 14-*O*-MeM6SU, 10 min after fentanyl injection and 30 min in the case of morphine injection (time of peak effect). Each value represents the mean in grams ± SEM. ^∗^*p* < 0.05 vs. diabetic baseline and saline treated group. #*p* < 0.05 vs. weight match control group (one way ANOVA followed by Newman–Keuls *post hoc* test).

Subcutaneous 14-*O*-MeM6SU (253, 506, and 1012 nmol/kg), fentanyl (25, 50, and 100 nmol/kg) and morphine (10000, 20000, and 40000 nmol/kg) were tested for their antiallodynic actions in diabetic rats with allodynia (Figure 4). 14-*O*-MeM6SU in all tested doses significantly ameliorated the allodynia (Figure 4A), whereas fentanyl and morphine only at a higher dose (100 and 40000 nmol/kg, respectively) attenuated the allodynia significantly (Figure 4B,C).

When we compared the effects of 14-*O*-MeM6SU and fentanyl or morphine doses in diabetic and non-diabetic rats, morphine in lower doses (from 2500 nmol/kg) induced significant antinociceptive actions in naïve (weight matched) rats. 14-*O*-MeM6SU at the 253 nmol/kg dose, which already produced antiallodynic effects in diabetic rats, failed to show any significant antinociceptive action in naïve rats. However, at higher doses (506 nmol/kg) it produced antinociception in naïve rats. Interestingly, fentanyl showed partial but significant antinociceptive effect in naïve rats in the doses of 25 and 50 nmol/kg, doses that failed to alter the thresholds of diabetic animals. This means that 14-*O*-MeM6SU, but not fentanyl or morphine did produce antiallodynic effects in certain doses devoid of antinociception in naïve rats. This effect might be attributed to the decrease in the opioid receptors, which in turn affects the action of test compounds but not that of 14-*O*-MeM6SU.

We further analyzed the lowest antiallodynic dose of 14-*O*-MeM6SU and morphine (253 and 40000 nmol/kg, respectively) at 12th weeks advanced diabetic rats. Both compounds produced antiallodynic effects in accordance with 9th week data at the same doses (data not shown).

### The Antagonist Effect of Coadministered NAL-M on the Systemic Antinociceptive Effect of 14-*O*-MeM6SU or Morphine in Diabetic Rats

**FIGURE 5:**
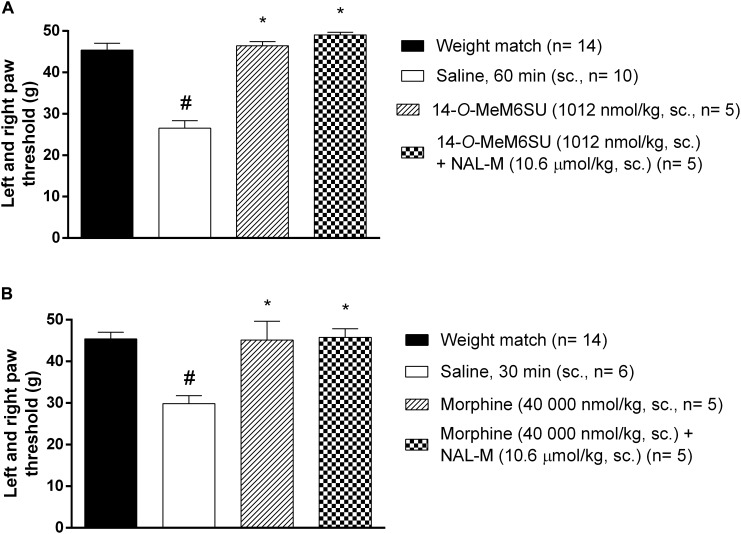
The antagonist effect of s.c. co-administered NAL-M (10.6 μmol/kg) on the analgesic effect of s.c. 14-*O*-MeM6SU **(A)** and morphine **(B)** in STZ treated neuropathic animals in doses that reversed the allodynia and elevated PPT on diabetic and non-diabetic animals. Data were obtained 60 min after the injection of 14-*O*-MeM6SU and 30 min in the case of morphine injection. Each value represents the mean in grams ± SEM. ^∗^*p* < 0.05 vs. diabetic baseline and saline treated group. #*p* < 0.05 vs. weight match control group (one way ANOVA followed by Newman–Keuls *post hoc* test).

The antagonist effect of the peripherally acting opioid antagonist NAL-M (10.6 μmol/kg, s.c.) was tested against s.c. 14-*O*-MeM6SU and morphine doses producing antiallodynic effects. In these experiments NAL-M failed to alter the antiallodynic action of test compounds (Figure 5), indicating the contribution of the central nervous system. NAL-M alone had no effect (*n* = 5, data not shown).

### Persistent Hyperglycemia Reduced MOR Immunoreactivity in Spinal Cord and DRG as Well as Binding Sites in the Spinal Cord of Rats

**FIGURE 6:**
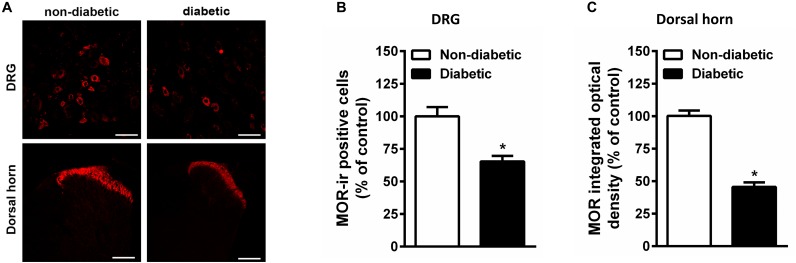
The immunohistological assay shows reduction in MOR number in DRG **(A,B)** and spinal cord dorsal horn tissues **(A,C)** of STZ treated diabetic rats in comparison with non-diabetic animals (*n* = 5). Scale bar = 20 μm for DRG and 40 μm for spinal cord section. Each value represents the mean ± SEM. ^∗^*p* < 0.05 Student *t*-test.

Constant hyperglycemia resulted in apparent decrease in the number of MOR positive DRG neurons in rats developed allodynia (Figure 6). In parallel, there is apparent reduction in the MOR immunoreactivity within superficial layer of dorsal horn in spinal cord of diabetic rats (Figure 6).

**FIGURE 7:**
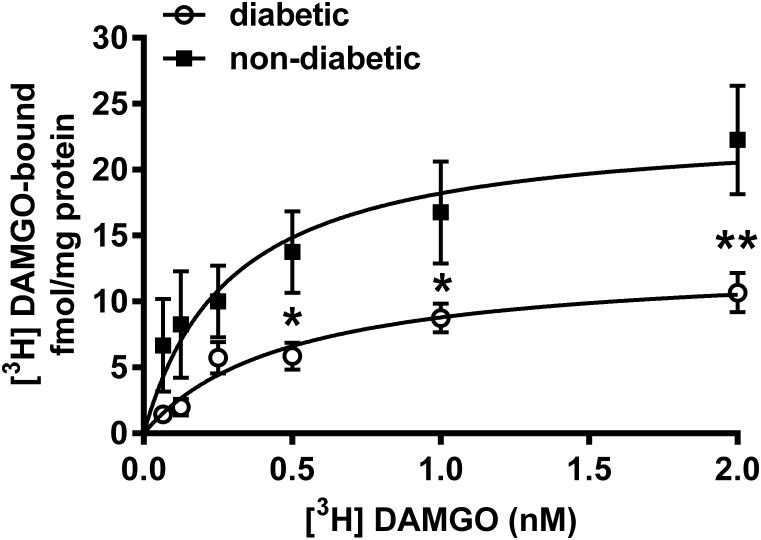
[^3^H]DAMGO binding in membrane tissues from dorsal spinal cord of diabetic and non-diabetic rats (*n* = 3–5). ^∗^*p* < 0.05; vs. non-diabetic control group (^∗∗^*p* < 0.01). (Two-way ANOVA followed by Fisher’s LSD *post hoc* test.)

Indeed, the radioligand binding assay demonstrated that the maximal of [^3^H]DAMGO by membrane spanning MOR (*B*_max_) was significantly decreased in the dorsal horn of diabetic rats (13.11 ± 1.85 fmol/mg) compared to controls (23.55 ± 4.36 fmol/mg) (*P* < 0.001; Figure 7). The dissociation constant (Kd) was 0.49 ± 0.18 for diabetic and 0.29 ± 0.17 for control rats. These data indicate no significant difference between diabetic and control rats in the affinity of DAMGO for MOR.

### The G-Protein Coupling Activity in Presence of 14-*O*-MeM6SU, Fentanyl or Morphine in Spinal Cord Homogenates Prepared From Diabetic or Control Rats

**FIGURE 8:**
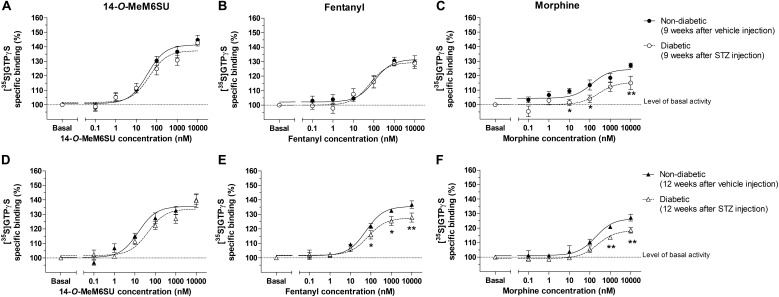
Agonist activity of 14-*O*-MeMSU **(A,D)** compared to fentanyl **(B,E)** and morphine **(C,F)** in rat whole **spinal cord** membrane homogenates treated with vehicle or STZ for 9 **(A–C)** or 12 weeks **(D–F)** after treatment in [^35^S]GTPγS binding assays. Figures represents the specific binding of [^35^S]GTPγS in the presence of increasing concentrations (0.1 nM–10 μM) of the indicated ligands. Points represent means ± S.E.M. for at least three experiments performed in triplicate. “Basal” on the *x*-axis indicates the basal activity of the monitored G-protein, which is measured in the absence of the compounds and also represents the total specific binding of [^35^S]GTPγS. The level of basal activity was defined as 100% (indicated by dotted line). The calculated *E*_max_ and EC_50_ ± S.E.M. values are presented in Table 1. ^∗^*p* < 0.05 diabetic vs. non-diabetic samples (^∗∗^*p* < 0.01; Two-way ANOVA, Fisher’s LSD *post hoc* test).

MOR specific G-protein coupling was measured by MOR agonist-stimulated [^35^S]GTPγS binding assay. 14-*O*-MeM6SU produced similar G-protein coupling in spinal cord tissues from STZ or vehicle treated rats after 9 or 12 weeks (Figure 8). On the other hand, fentanyl and morphine showed a significantly reduced efficacy (*E*_max_) of G-protein coupling in spinal cord tissues of diabetic rats. The *E*_max_ of morphine showed a significant decrease in the samples of 9th and 12th week, whereas in the case of fentanyl the decrease was apparent in the 12th week’s samples, in accordance with our previous data (Shaqura et al., 2013). The calculated *E*_max_ values for test compounds are presented in Table 1. The reduction in [^35^S]GTPγS specific binding of morphine and fentanyl was also observed between certain concentration points of the concentration-response curves (Figure 8). In general, 14-*O*-MeM6SU and fentanyl showed significantly higher efficacy than morphine in all of the spinal cord samples as indicated by the *E*_max_ values (Table 1). Taken together, no difference exists in 14-*O*-MeM6SU-stimulated coupling but it does exist in fentanyl- and morphine-stimulated coupling between diabetic and control rats.

**Table 1:** Maximum G-protein efficacy (*E*_max_ ± SEM) and potency (EC_50_ ± SEM) of 14-*O*-MeM6SU, compared to morphine and fentanyl in vehicle (non-diabetic) and 9 and 12 weeks STZ treated (diabetic) rat spinal cord performed in [^35^S]GTPγS binding assays.

	*E*_max_± SEM (%)		EC_50_± SEM (nM)	
		
	Non-diabetic	Diabetic	Non-diabetic	Diabetic
*9 weeks*				
14-*O*-MeM6SU	141.4 ± 2^###^ (*n* = 6)	137.3 ± 2.47^###^ (*n* = 5)	35.08 ± 11.75 (*n* = 6)	43.35 ± 19.94 (*n* = 5)
Fentanyl	131.6 ± 2.55^###^^+^ (*n* = 5)	129.5 ± 2.33^###^^+^ (*n* = 6)	63.94 ± 41.93 (*n* = 5)	53.95 ± 36.81 (*n* = 6)
Morphine	124.4 ± 2.09 (*n* = 7)	115.4 ± 2.97^∗∗^ (*n* = 7)	N.D.^1^	N.D.^1^
*12 weeks*				
14-*O*-MeM6SU	135.5 ± 1.88^##^ (*n* = 6)	133.8 ± 2.4^###^ (*n* = 5)	14.96 ± 5.66 (*n* = 6)	43.45 ± 21.17 (*n* = 5)
Fentanyl	135.5 ± 1.25^##^ (*n* = 6)	128.1 ± 1.94^##+∗∗^ (*n* = 6)	56.89 ± 12.9 (*n* = 6)	78.7 ± 34.71 (*n* = 6)
Morphine	126.6 ± 2.12 (*n* = 4)	118.3 ± 1.41^∗∗^ (*n* = 6)	N.D.^1^	N.D.^1^


*Values were calculated according to Figure 8. ^∗^Indicates the significant difference in diabetic samples compared to control (^∗∗^*P* < 0.01). ^+^Indicates the significant difference compared to 14-*O*-MeM6SU within control or diabetic samples (^+^*P* < 0.05). ^#^Indicates the significant difference compared to morphine within control or diabetic samples (^##^*P* < 0.01; ^###^*P* < 0.001). ^*1*^Not determined, since the EC_*50*_ values could not be interpreted. Two-way ANOVA followed by Fisher’s LSD *post hoc* test.*

### The G-Protein Coupling Activity in Presence of 14-*O*-MeM6SU, Fentanyl or Morphine in Brain Homogenates Prepared From Diabetic or Control Rats

**FIGURE 9:**
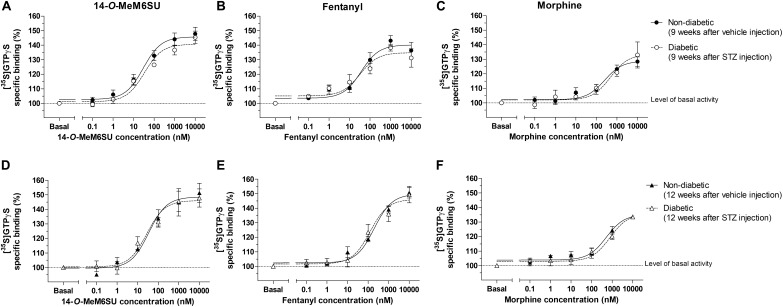
Agonist activity of 14-*O*-MeMSU **(A,D)** compared to fentanyl **(B,E)** and morphine **(C,F)** in rat whole **brain** membrane homogenates treated with vehicle or STZ 9 weeks **(A–C)** or 12 weeks **(D–F)** after treatment in [^35^S]GTPγS binding assays. Figures represents the specific binding of [^35^S]GTPγS in the presence of increasing concentrations (0.1 nM–10 μM) of the indicated ligands. Points represent means ± S.E.M. for at least three experiments performed in triplicate. “Basal” on the *x*-axis indicates the basal activity of the monitored G-protein, which is measured in the absence of the compounds and also represents the total specific binding of [^35^S]GTPγS. The level of basal activity was defined as 100% (indicated by dotted line). The calculated *E*_max_ and EC_50_ ± S.E.M. values are presented in Table 2.

MOR G-protein coupling in the presence of 14-*O*-MeM6SU, fentanyl or morphine was also determined in brain membrane homogenates from STZ or vehicle treated rats. Neither compounds showed significant differences in maximal efficacy (E_max_) and ligand potency (EC_50_) 9 or 12 weeks after STZ treatment compared to control samples (Table 2 and Figure 9). Additionally, in the control brain samples, 14-*O*-MeM6SU and fentanyl showed significantly higher maximum efficacy compared to morphine, whereas in the STZ treated brain samples of the 9^th^ week this significance disappeared, though the tendency remained (Table 2 and Figure 9).

**Table 2:** Maximum G-protein efficacy (*E*_max_ ± SEM) and potency (EC_50_ ± SEM) of 14-*O*-MeM6SU, compared to morphine and fentanyl in vehicle (non-diabetic) and 9 and 12 weeks STZ (diabetic) treated rat brain performed in [^35^S]GTPγS binding assays.

	*E*_max_ ± SEM (%)		EC_50_ ± SEM (nM)	
		
	Non-diabetic	Diabetic	Non-diabetic	Diabetic
*9 weeks*			
14-*O*-MeM6SU	145.7 ± 2.4^###^ (*n* = 6)	140.7 ± 2.1 (*n* = 6)	29.51 ± 11.44 (*n* = 6)	33.19 ± 19.09 (*n* = 6)
Fentanyl	140.2 ± 2.67^##^ (*n* = 7)	134.8 ± 2.84 (*n* = 5)	36.64 ± 18.42 (*n* = 7)	32.02 ± 20.14 (*n* = 5)
Morphine	128.8 ± 2.65 (*n* = 7)	133.5 ± 4.85 (*n* = 6)	N.D.^1^	N.D.^1^
*12 weeks*			
14-*O*-MeM6SU	148.5 ± 3.59^##^ (*n* = 4)	146.1 ± 3.22^#^ (*n* = 5)	34.99 ± 17.74 (*n* = 4)	27.86 ± 13.87 (*n* = 5)
Fentanyl	149.7 ± 2.38^##^ (*n* = 5)	146.1 ± 3.51^#^ (*n* = 4)	197.7 ± 54.05^+^ (*n* = 5)	124.74 ± 55.64 (*n* = 4)
Morphine	135.2 ± 2.65 (*n* = 4)	135.6 ± 3.08 (*n* = 4)	N.D.^1^	N.D.^1^


*Values were calculated according to Figure 9. ^#^Indicates the significant difference compared to morphine within control or diabetic samples (^#^*P* < 0.05; ^##^*P* < 0.01; ^###^*P* < 0.001). ^+^indicates the significant difference compared to 14-O-MeM6SU within control or diabetic samples (^+^*P* < 0.05). ^*1*^Not determined, since the EC_*50*_ values could not be interpreted. Two-way ANOVA followed by Fisher’s LSD *post hoc* test.*

## Discussion

In the present study, we have examined the opioid antinociceptive-antiallodynic efficacy in the mitigation of rat neuropathic pain by three MOR agonists, namely, 14-*O*-MeM6SU, fentanyl and morphine. The test compounds display different pharmacological character in term of the efficacy (Lacko et al., 2012; Shaqura et al., 2013). The analgesic effects of currently available opioid analgesics in the management of neuropathic pain is a matter of controversy in both clinical practice and opioid research. Nevertheless, opioids and a related compound, tramadol are considered as second line agents in the management of painful diabetic neuropathy (Moulin et al., 2014). It is noteworthy to mention that opioids in painful conditions like NP due to cancer, or acute NP, are considered as first line agents, and may also be useful to achieve fast pain relief during titration of the conventional first line medications (Dowell et al., 2016). The majority of opioids available for clinical use produce their analgesic effects by activation of MOR. In animal models of diabetes, the MOR reserve has been reported to be reduced in rats of 2–3 weeks diabetes (Kou et al., 2016). Herein, we paid attention to the impact of persistent hyperglycaemia (9–12 weeks) on MOR-mediated antinociception by 14-*O*-MeM6SU, fentanyl and morphine in rats. To the best of our knowledge, this is the first work that reports on the antiallodynic effect of the recently synthetized opioid agonist of high efficacy, 14-*O*-MeM6SU compared to that of opioids of high clinical values, fentanyl and morphine on DNP in rats following systemic administration (Lacko et al., 2012).

The first task was to follow the changes in blood glucose level and development of allodynia for 12 weeks period following STZ-treatment. STZ is a diabetogenic drug commonly used for inducing diabetes. It’s diabetogenic action is a result of β-cell destruction as proved by our and other previous works (Courteix et al., 1993, 1994; Shaqura et al., 2013). In the present work animals with (≥14 mmol/l) blood glucose level were considered diabetics as described previously (Courteix et al., 1993). We have measured significant allodynia 3 weeks following STZ treatment that peaked at the 9th-12th week (Figure 2). These symptoms are indicative for development of DNP and in accordance with previous studies (Courteix et al., 1993; Goyal et al., 2016).

In addition other symptoms related to development of diabetes such as polydipsia, polyphagia and weight change were also assessed. In this series of experiment the latter parameters were only determined during the first 7 weeks following STZ injection (Supplementary Figure 1). To carry out such experiments animals were kept individually. The assessment of these parameters was ended on the 7th week. The purpose was to exclude the isolation stress of the rats when we assessed analgesia. Generally, rodents prefer some form of group living. It is well known that stress is significantly reduced in rats that are housed in groups compared with rats housed alone (Murínová et al., 2017). We also paid attention to the impact of diabetes in gastrointestinal function. Although both acute and chronic hyperglycemia can impair the gastrointestinal motility (Horowitz et al., 2002), in this study the rate of gastric emptying was comparable in diabetic and non-diabetic rats, suggesting that gastroparesis did not develop in spite of constantly high blood glucose level.

Despite of a huge research, there is no present data that could end the debate on the controversy of effectiveness of current opioid analgesics in the management of DNP. In addition, only a few studies were carried out on the antinociceptive-antiallodynic effects of opioids at advanced diabetes of 9–12 weeks that at least could suit the clinical condition of advanced neuropathic pain. To carry on, we examined the change in the antinociceptive effects of the test compounds (14-*O*-MeM6SU, fentanyl and morphine) in both naïve (weight match control) and diabetic rats at 9 weeks after STZ-treatment. In addition to the drug’s antinociception, the antiallodynic actions of the test compounds were also determined. Keeping in mind, when we explain the antinociception, we refer to the change in the paw thresholds of control and diabetic rats. On the other hand, when speak about antiallodynic effects, we refer to the effects of test compounds that only seen in rats with hyperglycemia that developed tactile allodynia. Impairment in the antinociceptive effect was seen only for systemic morphine. This tendency is based on the calculated ED_30_ values where morphine was 7 times less effective in diabetic animals than non-diabetic ones, whereas fentanyl or 14-*O*-MeM6SU showed no difference in the antinociceptive action between diabetic and non-diabetic animals (Figure 3, ED30_diabetic_/ED30_non-diabetic_). This indicates a significant reduction in the antinociceptive effect of morphine, which is in accordance with previous studies (Courteix et al., 1998; Cegielska-Perun et al., 2014). Yet, the novel compound and fentanyl remained highly effective even in neuropathic conditions.

Regarding to antiallodynic effect, our study is new from two aspects. The first aspect is the novel compound but not fentanyl or morphine at the present work circumstances produced antiallodynic effect in doses devoid of an impact on naïve rats. This statement is based on the following observations: 14-*O*-MeM6SU (253 nmol/kg) but not fentanyl or morphine produced significant antiallodynic action only in DNP and no impact on PPTs of naive rats.

Previous studies by our and other groups reported on the effects of different opioids in diabetic NP, though the antiallodynic efficacy was varied (Courteix et al., 1998; Shaqura et al., 2013, 2014; Rutten et al., 2014). Many studies reported on the lowered opioid antinociceptive efficacy in animal neuropathic models (Cegielska-Perun et al., 2014; Shaqura et al., 2014; Yadlapalli et al., 2016). Some studies reported the ineffectiveness of morphine even in doses up to 10 mg/kg (approximately 31 μmol/kg) 7 weeks following STZ treatment (Yamamoto et al., 2009). Of note, in contrast to morphine, 14-*O*-MeM6SU or fentanyl displayed equipotent antinociception in non-diabetic and diabetic rats.

We further analyzed the peripheral antiallodynic component of morphine and 14-*O*-MeM6SU in the presence of systemically given NAL-M, because in our previous works, in inflammatory pain models 14-*O*-MeM6SU in certain doses showed peripheral antinociception (Lackó et al., 2016; Balogh et al., 2018). The applied NAL-M dose has been reported previously for its peripheral distribution (Bianchi et al., 1982; Lewanowitsch and Irvine, 2002). Under the present experimental circumstances, systemic NAL-M failed to affect the antiallodynic effect of systemic 14-*O*-MeM6SU or morphine (Figure 5). Consequently, if we accept that NAL-M does not penetrate the blood brain barrier in the applied doses, then, MOR in CNS might mediate the measured antinociceptive effect of higher systemic doses of test compounds that abolished allodynia, in accordance with previous studies (Zurek et al., 2001). A question might be raised about the peripheral vs. central antiallodynic effect of fentanyl. Regarding to this issue fentanyl has physiochemical properties that allow it to readily access to the CNS following systemic administration as described by previous studies (Fürst et al., 2005). Therefore, involvement of central opioid system in the antiallodynic effect of fentanyl is not a matter of debate.

The central opioid analgesic effect is originated from the activation of opioid receptors in the spinal and supraspinal region as described elsewhere (Pasternak, 1993). The analysis of MOR functioning at both spinal and supraspinal levels was carried out to elucidate the above mentioned and stand for our second aspect.

The second aspect is that, at the level of spinal cord, 14-*O*-MeM6SU or fentanyl but not morphine caused remarkable effect in G-protein coupling in spinal tissues prepared from rats with DNP 9 weeks following STZ treatment (Figure 8 and Table 1). Further extension of blood hyperglycemia impaired both morphine and fentanyl induced MOR activation, though the degree of impairment in the case of morphine was prominent.

In our previous work in rats with advanced (12 weeks) diabetic neuropathy and mechanical hyperalgesia, we demonstrated a decrease in fentanyl-mediated spinal antinociception in mechanical hyperalgesia associated with reduced MOR number and G-protein coupling in sensory neurons (Shaqura et al., 2013). In the present work we also detected a decrease in MOR density both in the DRG and the dorsal horn of the spinal cord of rats with DNP (Figure 6). On the other hand, we have previously proved that 14-*O*-MeM6SU has higher intrinsic efficacy than morphine (Khalefa et al., 2012), meaning that even if there is a decrease in MOR reserve it might activate MOR and produce measurable analgesia.

The results presented herein proved that 14-*O*-MeM6SU and fentanyl are opioids of high efficacy, because they could produce significant G-protein activation in spinal cord homogenates from diabetic rats after 9-12 of STZ treatment. In contrast to 14-*O*-MeM6SU or fentanyl, morphine displayed very weak G-protein activation at the same time points. Of note, fentanyl showed significantly lower efficacy in all spinal samples compared to the novel compound but was superior to morphine. On the other hand, neither morphine nor 14-*O*-MeM6SU or fentanyl showed any difference in efficacy at the supraspinal level of diabetic rats compared to non-diabetic. Interestingly, 14-*O*-MeM6SU and fentanyl, but not morphine, showed similar efficacy at the spinal level in control and diabetic rat after 9 weeks. Furthermore, activation of MOR by 14-*O*-MeM6SU was not affected by developed diabetes and remained significantly higher compared to the other test compounds. Our previous study showed reduction in the analgesic action of fentanyl in diabetic rats of 12 weeks. The impairment of fentanyl analgesia was related to decrease in MOR functioning, as described previously (Shaqura et al., 2013). At the present we can relate the difference in G-protein coupling property of brain compared to the spinal cord to the difference in their MOR reserve. It means that the magnitude of action largely depends on the efficacy (intrinsic activity) of test opioid agonists. Morphine has been reported to display lower efficacy (partial agonist) either in isolated organs hosting opioid receptors or G-protein activation assay (Rónai et al., 2006; Al-Khrasani et al., 2007). In contrast to morphine, 14-*O*-MeM6SU or fentanyl were found to be full agonists (higher intrinsic activity) (Lacko et al., 2012; Khalefa et al., 2013). Therefore, if the receptor reserve is decreased then only opioids of high efficacy could show activity regardless of circumstances *in vitro* or *in vivo*. Herein, the density of opioid receptors was lower at the spinal level and no change was observed in the brain of diabetic rats. In addition, MORs are distributed in the pre- and postsynaptic membranes of primary and secondary sensory neurons, respectively, that convey pain toward the brain. Consequently, this condition (low spinal MOR density) could affect the spinal analgesic action of low efficacy opioids such as morphine.

[35S]GTPγS binding assays are used to demonstrate the alterations in the G-protein activity in the presence of opioid agonists. The activation of G-protein is the initial step of the GPCR signaling pathway. Indeed, this activity was disrupted in MOR receptors expressed in the spinal cord (see Results section: The G-protein Coupling Activity of 14-*O*-MeM6SU, Fentanyl or Morphine in Spinal Cord Homogenates Prepared From Diabetic or Control Rats), which corresponds well with results seen in immunohistochemistry and saturation binding experiments. Signaling mechanism of morphine has been reported to engage both G-protein coupling and β-arrestin recruitment. However, at the present time we have no data on the mechanism of 14-*O*-MeM6SU regarding to β-arrestin recruitment. Future studies are needed to explore whether 14-*O*-MeM6SU is biased to G-protein or β-arrestin.

The spinal cord is a crucial point in pain transduction (Pasternak, 1993). At this pain traffic point MORs are found in the presynaptic central terminals of primary afferent neurons, which are the targets for spinally administered opioids and other drugs prescribed for NP, like gabapentinoids (Perret and Luo, 2009). These analgesic agents block the voltage gated calcium channels (VGCCs), and consequently transmitters that further process pain toward the brain. Since opioid receptors are localized in both pre- and postsynaptic membrane of primary afferent and secondary afferent fibers, respectively their activation will result in the inhibition of transmitter release and consequently the peripheral signal propagation toward the brain.

Therefore, we can hypothesize that 14-*O*-MeM6SU, a high efficacy opioid indicated by high G-protein coupling, might block the pain effectively at this point. Under the present experimental conditions, morphine did produce weak G-protein coupling in spinal homogenates from diabetic rats, so behaved as a partial agonist, meaning that at higher doses its analgesic action might be stemmed from the activation of opioid receptors at the supraspinal level rather than opioid receptors at the presynaptic site of central terminal of primary afferent. MOR number apparently was low at the level of DRG and spinal dorsal horn, in accordance with previous data that showed a significant decrease in MOR in the spinal cord of animals with diabetic neuropathy (Shaqura et al., 2013). Therefore, the supraspinal region is largely responsible for morphine analgesia supported by G-coupling in the present work and other *in vivo* studies (Muranyi and Radak, 2008; Al-Khrasani et al., 2012). In addition, advance in diabetes (12 weeks) could also affect the effect of fentanyl on G-protein activation, though in contrast to morphine it still produces significant effect in term of efficacy (*E*_max_). An important question might be raised on the impact of test compounds on the respiratory functions. In our previous work 14-*O*-MeM6SU in a dose of 253 nmol/kg showed no respiratory depression, yet in the present work could produce significant antiallodynic action. On the other hand morphine or fentanyl produced antiallodynic effects only in higher doses that are well known to cause significant respiratory depression (Pazos and Flórez, 1984; Hutchinson et al., 2008; Cavalla et al., 2015). Taken together, the present study established the ineffectiveness of morphine and in contrast to 14-*O*-MeM6SU proved change on G-protein coupling between the 9th and 12th week of diabetes in the case of fentanyl. Future studies are needed to explore the role of spinal opioid receptors compared to supraspinal ones in the inhibition of DNP. Of note, in the present work we did our best to obtain our conclusion from one species (rats) applying *in vivo* and *vitro* assays. The *in vivo* results were supported by G-protein coupling and immunohistochemical assays. G-protein activation is undoubtedly an important step to establish the response of the cells to ligands. Immunohistochemistry is an important tool to assess the change of tissue-based protein expression. Therefore, the outcome of the present study hopefully will contribute to better understanding the mechanisms underlying the variation in the response of patients as well as animals with DNP to current opioids.

In the light of above mentioned, the present study also could shed light on the efficacy of opioids in the management of advanced diabetes (9 and 12 weeks) applying three different opioid agonists: one novel and two are considered as classical opioids of clinical values. Of note the test compounds display different physiochemical profiles, efficacy and duration of actions. Also proved that only diabetes of 12 weeks long could discriminate between opioid analgesics of high efficacy that had shown similarity in 9 weeks or earlier stage of diabetes.

## Conclusion

Large reduction in the antinociception of morphine but not of fentanyl or 14-*O*-MeM6SU in diabetic rats compared to control rats was observed.

Alterations in the antinociceptive effects of morphine and fentanyl but not 14-*O*-MeM6SU were shown in diabetic neuropathic rats.

Untreated diabetes results in reduced MOR G-protein coupling by morphine and fentanyl but not 14-*O*-MeM6SU at the level of spinal cord, key traffic point in the pain transmission.

Advanced diabetes results in significant reduction in the antiallodynic effects of partial agonists like morphine in contrast to the opioid agonists with high efficacy: 14-*O*-MeM6SU or fentanyl. Developing novel opioids with high efficacy in the management of advanced painful diabetes is of unmet medical needs.

## Ethics Statement

All housing and experiments were performed in accordance with the European Communities Council Directives (2010/63/EU), the Hungarian Act for the Protection of Animals in Research (XXVIII.tv. 32.§) and local animal care committee (PEI/001/276-4/2013).

## Author Contributions

MA-K, SB, KG, and SF participated in the research design. MB, ZZ, MoS, SAM, KK, AM, BV, BL, and FZ conducted the experiments. SH contributed new reagents or analytic tools. MB, FZ, and PR performed data analysis. MA-K, MS, ZZ, and MB wrote or contributed to the writing of the manuscript.

## Conflict of Interest Statement

The authors declare that the research was conducted in the absence of any commercial or financial relationships that could be construed as a potential conflict of interest.

## Supplementary Material

Figure S1 Daily food and water consumption of STZ treated (diabetic) and vehicle treated (non-diabetic) animals at the given time points.

Figure S2 Time dependent effect of 14-*O*-MeM6SU and morphine in diabetic animals with developed allodynia. 14-*O*-MeM6SU and morphine achieved their peak effects at 60 and 30 min, respectively.
