# Supplementary material for: Efficacy-Based Perspective to Overcome Reduced Opioid Analgesia of Advanced Painful Diabetic Neuropathy in Rats
Source: Front Pharmacol. 2019 Apr 9;10:347. doi: 10.3389/fphar.2019.00347 (PMC6465774; doi:10.3389/fphar.2019.00347)
Supplement: Supplementary file 3 [file Data_Sheet_1.docx]

**Supplementary Fig. 1.:** Daily food and water consumption of STZ treated (diabetic) and vehicle treated (non-diabetic) animals at the given time points.

**Supplementary Fig. 2.:** Time dependent effect of 14-*O*-MeM6SU and morphine in diabetic animals with developed allodynia. 14-*O*-MeM6SU and morphine achieved their peak effects at 60 and 30 min, respectively.
